# MiR-124 suppression in the prefrontal cortex reduces depression-like behavior in mice

**DOI:** 10.1042/BSR20190186

**Published:** 2019-09-13

**Authors:** Zhiwen Gu, Jiyang Pan, Liping Chen

**Affiliations:** 1 Department of Psychiatry, The First Affiliated Hospital, Jinan University, Guangzhou City 510630, P.R. China; 2 Department of Psychiatry, Guangzhou First People’s Hospital, School of Medicine, South China University of Technology, Guangzhou City, Guangdong Province 510180, P.R. China

**Keywords:** anti-depressive, depression, miR-124, molecular regulation, prefrontal cortex

## Abstract

Depression is a potentially life-threatening mental disorder with unknown etiology. Several microRNAs (miRNAs) have been shown to play critical roles in the etiology of depression. Here, we aim to elucidate the anti-depressive behavior of miR-124 suppression in prefrontal cortex (PFC). Quantitative real-time PCR (RT-PCR) was used to evaluate the expression of miR-124 and SIRT1 in the PFC of a chronic unpredictable mild stress (CUMS) model. The PFC of C57BL/6J mice was bilaterally injected with lentiviral vectors (LV) for ectopic expression of SIRT1, miR-124, or miR-124 inhibitor (si-miR-124). The anti-depressive behavior was observed after injection of LV-SIRT1 or LV-si-miR-124 into the PFC, using behavior tests including latency to feed, food and water intake, sucrose preference test, and forced swimming test. MiR-124 overexpression and inhibition resulted in upregulation and down-regulation of SIRT1 and cyclic AMP responsive element binding protein 1 (CREB1), respectively. MiR-124 overexpression exacerbated depression-like behaviors and decreased SIRT1. Further, dual-luciferase assay confirmed that SIRT1 was a target of miR-124. Taken together, a potential molecular regulation of miR-124 on SIRT1 is revealed by our study and miR-124 suppression in PFC is a potential strategy to reduce depression-like behavior.

## Introduction

Depression is considered as one of important prevalent disorders of psychiatry throughout the world and is the important concern for public health [1,2]. Unfortunately, a large portion of patients fail to respond to existing anti-depressive interventions [3,4], partially due to the lack of understanding of molecular pathophysiology of depression. Evidences suggested that depression may arise from environmental and genetic factors [5]. Therefore, new therapeutic strategies against depression are still in urgent need.

Recently, a class of non-coding RNAs, microRNAs (miRNAs), have emerged as important regulators of the higher functioning of brain [6,7]. MiRNAs bind to 3′ untranslated regions (3′-UTR) of the target mRNA for suppressing target gene expression in a post-transcriptional manner [8]. MicroRNA-124 (miR-124) is expressed abundantly and specifically in all regions of the brain except the pituitary gland. MiR-124 expression is approximately 100 times lower in all other tissues [9,10]. MiR-124 has been demonstrated to participate in the key processes of central nervous system (CNS) involved the neuronal development, synaptic plasticity and neurogenesis [11]. Disruptions in such processes was shown linked to the development of depression [12]. Recent studies using animal models of depression suggested that dysregulation in sirtuin 1 (SIRT1) gene expression plays an important role in the depression-like behaviors [13,14]. Furthermore, SIRT1 is recently implicated in the mood disorders of mice [15] as well as humans [16], whereby the reduction in levels of SIRT1 in blood has been linked to the depressive behaviors. A number of signaling pathways, including Akt/GSK3β signaling pathway [17], mTOR signaling [18], etc. are found to be mediated by SIRT1, posing SIRT1 as a potential mediator of neuron growth and development of depression-like behaviors. Therefore, SIRT1 is frequently pursued as a target for treatment of depression, whereby miRNAs, which are broad-spectrum regulators of genes, have been shown to regulate SIRT1 expression [19,20].

In the present study, we aim to explore the role of miR-124 in depression and evaluate the efficacy of miR-124 suppression in attenuating depression. We demonstrated that miR-124 upregulation is correlated with decreased SIRT1 expression in the prefrontal cortex (PFC) of a chronic unpredictable mild stress (CUMS) model. miR-124 suppression was effective in reducing depression-like behaviors of the CUMS model. SIRT1 was shown to be a target of miR-124. This inverse correlation between the miR-124 and SRT1 expression expands our understanding of the etiology of depressive disorders and potentiated the use of miR-124 suppressive as an anti-depressive strategy.

## Materials and methods

### Animals

C57BL/6J mice, which were acquired from Beijing Vital River Laboratory (China), were housed in a SPF room maintained with a 12h light/dark schedule, ambient temperature (22 ± 2°C) and 55 ± 5% relative humidity. The animals acclimatized to the housing conditions for a week before experiments. The animals were given free access to standard chow and water. All procedures were approved by the Institute for Experimental Animals of The First Affiliated Hospital, Jinan University.

### Chronic unpredictable mild stress protocol

Briefly, mice in CUMS was induced in mice using the following stressors: 24 h water deprivation, 24 h food deprivation, 5 min cold swimming (at 6°C), physical restraint for 2 h, 1 min tail pinch (1 cm from the end of the tail), exposure to mouse odor (removal of the cage containing the experimental mice from the procedure room and placing the experimental mice into cages in which cats had been held) for 1 h and overnight illumination. For a duration of 5 weeks, one of these stressors (in random order) was implemented daily. Mice in the control group were housed under identical conditions in a separate room with no contact with the stressed animals. Behavioral test was performed before mice were killed.

### Lentivirus vectors

SIRT1 lentiviral vector (LV-SIRT1) construction and subsequent lentivirus production were completed by Gene Pharma Company (Shanghai, China). The empty vector (LV-Mock) was used as a negative control. LV-miR-124 (108 TU/ml) and LV-si-miR-124 (108 TU/ml) were also purchased from the Gene Pharma Company (Shanghai, China).

### Stereotaxic injection of LV-Mock, LV-SIRT1, LV-miR-124, and LV-si-miR-124

Lentiviral injection was performed at 1 week before model construction. Mice were anesthetized with a ketamine/xylazine mixture (100 and 10 mg/kg, respectively, i.p.) and a stereotaxic frame was installed. Bilateral craniotomies were performed using a 0.5 mm diameter drill, targeted bilaterally to the PFC using the following coordinates: AP, +2.8 mm from bregma; ML, ±0.5 mm; and DV, −0.3 to −0.1 mm from the skull surface. Prior to injection, the needle was lowered to the target site and remained still at the position for 2 min. Lentivirus solutions were slowly and continually injected at 0.2 μl/min and a total volume of 1 μl was injected using a glass micropipette attached to the stereotaxic apparatus. After injection, the needle stayed in the same spot for 5 min before withdrawing. Subsequently, mice were kept warm until full recovery from anesthesia. The mice were allowed to recover for at least 1 week before behavioral experiments were conducted. Mice recovered for 1 week before exposure to CUMS.

### Depression-like behavioral tests

#### Novelty-suppressed feeding test

The NSFT was carried out in a plastic box (35 cm × 35 cm) at 5 weeks after CUMS exposure. Mice fasted 24 h before the test. The test was begun by placing a single pellet of food on a white paper platform at the center of the box. A mouse was placed in a corner of the maze and a stopwatch was used for timing. Timer was stopped when the mouse fetched the food using its forepaws and started eating. As a control, home-cage food consumption was measured within 90 min of the test’s completion.

#### Sucrose preference test

Mice were trained to adapt to a sucrose solution (1%, w/v) prior to the test. The adaptation was performed by placing two bottles of sucrose solution in cage for 1 day, followed by replacing one of bottles of sucrose solution with water. After another day, mice were deprived of water and food for 24 h. During the test, each mouse was housed in a cage, with free access to 1 bottle of sucrose solution and 1 bottle of water. After 24 h, the consumption of sucrose solution and water were documented.

#### Forced swim test

The forced swim test was performed at 24 h after the sucrose preference test. Mice were placed into a test beaker filled with tap water (22–26°C), and mice were unable to escape or rest by touching the bottom of the beaker. For 6 min, and the duration of mobility, which is defined as any movement beyond that necessary to maintain the head above water, was recorded. Data were expressed as the mean time of immobility, swimming, and climbing within the 6 min observation period.

### Primary cortical neuron cultures

After mice scarification using a CO_2_ chamber, cerebra cortices were carefully dissected. The neuronal cells were dissociated with trypsin (Gibco), re-suspended in a neurobasal medium (Gibco) (B27 19 final, Gibco) containing 2 mM GlutaMAX, and diluted to a concentration of 100 cells/ml. A total of 3 ml (approximately 300 cells) of cell suspension was added to each 35-mm poly-d-lysine-coated plate and cultured for 2 h in 5% CO_2_ at 37°C. The medium was then replaced with 3 ml of neurobasal/B27 medium, and cultured in 5% CO_2_ and 21% O_2_ for 2 weeks. After treatment with LV-miR-124 or LV-simiR-124 for 48 h, SIRT1 and CREB1 (cyclic AMP responsive element binding protein 1) protein expression levels were analyzed by Western blot.

### Quantitative real-time polymerase chain reaction

Quantitative real-time PCR (qRT-PCR) was performed using samples from mice with 5 weeks of stress (5 points) and their corresponding control samples. Total RNA was extracted from cells or tissues using the TRIzol agent (Invitrogen, Inc., Carlsbad, CA), followed by cDNA synthesis using the PrimeScript® RT Reagent Kit (Takara Co., Ltd., Dalian, China). qRT-PCR assay was performed to evaluate expression of mature miRNAs and mRNA using the SYBR Premix Ex TaqTM II (Takara Co., Ltd., Dalian, China). Expression levels of each sample were quantified using the 2^−ΔΔ*C*_t_^ method. The primers were implemented as follows: miR-124 forward primer (FW): 5′-GCGCTAAGGCACGCGGT-3′; reverse primer (RV): 5′-CAGTGCAGGGTCCGAGGT-3′. SIRT1 FW: 5′-CCCTTCTCAGTCTGCTCCAC-3′; RV: 5′-CTCCACGAACAGCTTCACAA-3′. U6 FW: 5′-CTCGCTTCGGCAGCACATATACT-3′; RV: 5′-ACGCTTCACGAATTTGCGTGTC-3′. GAPDH FW: 5′-ACCTTTGGCATTGTGGAAGG-3′, RV: 5′-ACACATTGGGGGTAGGAACA-3′. The relative levels of miRNA and mRNA were normalized to U6 snRNA and GAPDH, respectively.

### Western blot

Proteins of 40–50 μg isolated from cells or tissues were resolved by SDS/PAGE, followed by transferring to polyvinylidene difluoride membranes (PVDF; Bio-Rad, U.S.A.). The membranes were blocked with non-fat milk and then probed with antibodies against SIRT1 (ab110304, abcam), CREB1 (ab32515, abcam), p-CREB1 (ab32096, abcam), and GAPDH (ab181602, abcam). After washing, blots were incubated with horseradish peroxidase-conjugated secondary antibodies. GAPDH was used as a loading control.

### Luciferase assay

TargetScan analysis was used to identify binding regions between SIRT1 and miR-124. The possible target positions of the wild-type SIRT1 3′-UTR (WT-SIRT1) or mutated sequences (Mut-SIRT1) were inserted into the pmirGLO Dual-Luciferase miRNA target expression vector (Promega, Madison, WI). Luciferase assays were performed in 293 T cells using the Dual-Luciferase Reporter 1000 Assay System (Promega). Normalized values (firefly activity/Renilla activity) were used for analysis.

### Statistical analysis

All data were presented as the mean ± SD. Data were analyzed using *t*-tests and one-way analysis of variance (ANOVA). Bonferroni posttests were used when *F* values were significant, using GraphPad Prism 5 (GraphPad Software, Inc., La Jolla, CA, U.S.A.). The results were considered significant at *P*<0.05. Raw data of each figure and statistical analysis are shown in Supplementary Tables S1–S5.

## Results

### CUMS induces miR-124 up-regulation and SIRT1 down-regulation in PFC

**Figure 1:**
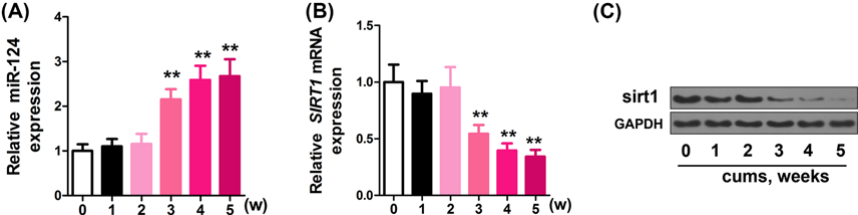
Up-regulation of miR-124 and reduction of SIRT1 in PFC induced by CUMS Alteration of miR-124 (**A**) and SIRT1 (**B,C**) expression PFC induced by CUMS over time points shown, protein levels of SIRT1 were provided with representative Western blot images, and quantitative analysis of SIRT1 protein levels normalized to GAPDH. **P<0.01.

To explore the effects of CUMS on miR-124 and SIRT1 levels, qRT-PCR was performed in PFC of mice that received CUMS. As shown in Figure 1, increasing duration of CUMS increased and decreased miR-124 (Figure 1A) and SIRT1 (Figure 1B) levels respectively (miR-124 and SIRT1 levels at 3–5 weeks compared with those at 0 week, *P*<0.001). To validate the change of sirt1 with CUMS, Western blot analysis was performed and the time-dependent down-regulation of sirt1 was also observed (Figure 1C).

### CUMS-induced depression-like behaviors can be altered by miR-124 overexpression or inhibition

**Figure 2:**
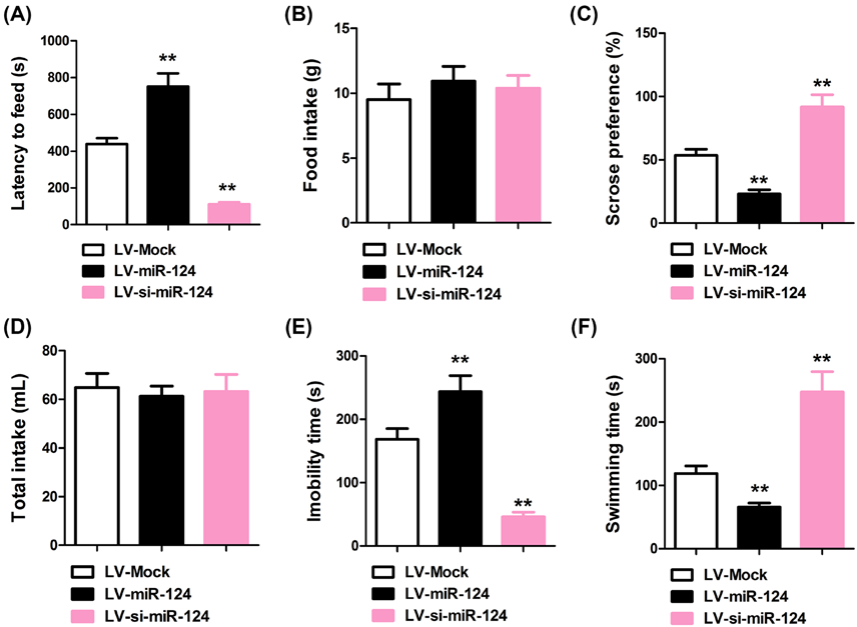
Effect of lentiviral-mediated miR-124 modulation on CUMS-induced depression-like behavior in the NSFT, SPT, and FST (**A**) Eating delay as measured by the latency to bite food in a novel anxiogenic environment following 24 h food deprivation. (**B**) The home-cage feed consumption within 90 min following the NSFT. (**C**) Sucrose preference was calculated by the following formula: preference = (sucrose solution intake/total fluid intake) × 100, and (**D**) total fluid intake (sucrose intake + water intake). (**E**) Immobility and (**F**) swimming in FST. *n*=8, **P<0.01 compared with LV-Mock group.

To further elucidate the role of miR-124 in CUMS induced depression, we overexpressed and inhibited miR-124 expression using lentiviral transfection in the PFC of mice that received CUMS. Latency to feed, food intake, sucrose preference, total intake, immobility time, and swimming time were used as parameters of behavior test to assess depression-like behaviors. As shown in Figure 2, compared with mice transfected with LV-Mock, mice with miR-124 overexpression demonstrated increased latency to feed (751 ± 71.79 s vs. 439 ± 32.02 s, *P*<0.001, *n*=8, two-tailed unpaired Student’s *t-*test, Figure 2A) and immobility time (242 ± 25.04 s vs. 170 ± 16.61 s, *P*<0.001, *n*=8, two-tailed unpaired Student’s *t-*test, Figure 2B), and decreased sucrose preference (22 ± 3.35% vs. 53 ± 5%, *P*<0.001, *n*=8, two-tailed unpaired Student’s *t*-test, Figure 2C) and swimming time (65 ± 6.28 s vs. 120 ± 11.65 s, *P*<0.001, *n*=8, two-tailed unpaired Student’s *t*-test, Figure 2D) (*P*<0.01), suggesting elevated depression. Inhibition of miR-124 exerted the opposing effects. No observable changes in food intake (Figure 2E) and total water intake (Figure 2F) were induced by miR-124 overexpression or suppression. These evidences are strong indicators that manipulation of miR-124 can greatly alter the depression-like behaviors induced by CUMS.

### miR-124 down-regulates SIRT1 in PFC

**Figure 3:**
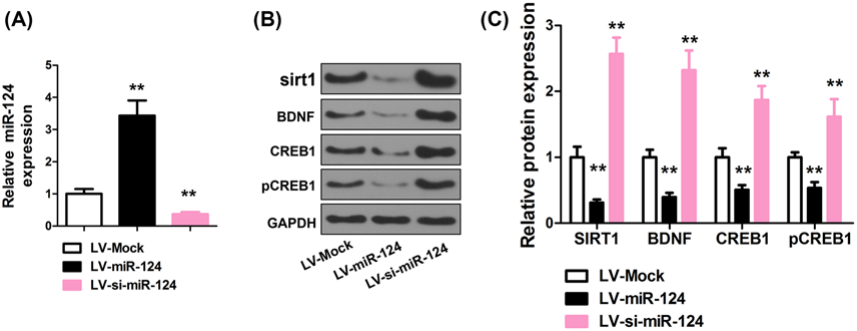
miR-124 down-regulates SIRT1 in PFC (**A**) Relative miR-124 level in PFC response to LV-miR-124 or LV-si-miR-124 treatment. miRNA levels were normalized to U6. (**B**) Effect of lentiviral-mediated miR-124 modulation on sirt1, BDNF, CREB1, and pCREB1 expression in PFC with representative Western blot images shown. (**C**) Quantitative analysis of SIRT1, BDNF, CREB1, and pCREB1 protein level normalized to GAPDH or CREB1. *n*=8. **P<0.01 compared with LV-Mock group.

Given the important role of SIRT1 gene in depression and the down-regulation of SIRT1 under CUMS, we further examined if SIRT1 expression is affected by miR-124. To this end, SIRT1 expression in PFC of mice, which were treated with lentiviruses for overexpression or down-regulation of miR-124 in PFC. The miR-124 levels increased 3.4 ± 0.47 folds after Lv-miR-124 transfection (*P*<0.001, *n*=8, two-tailed unpaired Student’s *t*-test) and decreased to 0.36 ± 0.07 of the normal level after LV-si-miR-124 transfection (*P*<0.001, *n*=8, two-tailed unpaired Student’s *t*-test, Figure 3A). As shown in Figure 3B,C, miR-124 overexpression and down-regulation led to suppression and induction of sirt1 levels, respectively, in PFC. A similar trend was observed for BDNF, CREB1, and pCREB1 levels (all *P*<0.001, *n*=8, two-tailed unpaired Student’s *t*-test, Figure 3B,C). Therefore, an antagonizing effect exist of miR-124 on SIRT1.

### SIRT1 is a functional target of miR-124

**Figure 4:**
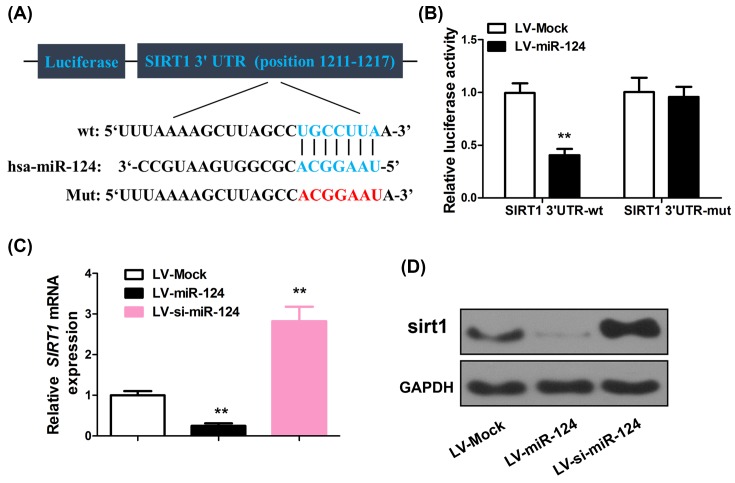
SIRT1 is a functional target of miR-124 (**A**) Specific locations of miR-124 binding sites in SIRT1 3′-UTR (upper and middle sequence), the ideograph of the mutated SIRT1 3′-UTR with seven seed nucleotide deletions were shown (bottom sequence). (**B**) Luciferase activity of the wild-type (WT) or mutant (MUT) SIRT1 3′-UTR reporter gene in 293 T cells with ectopic expression of miR-124. (**C,D**) SIRT1 alteration in the cultured primary cortical neurons after LV-miR-124 or LV-si-miR-124 treatment. **P<0.01 compared with LV-Mock group.

To clarify the interaction between miR-124 and SIRT1, TargetScan analysis was conducted, which identified a binding site between the wild-type SIRT1 3′-UTR and miR-124. Mutagenesis was induced in the binding region, yielding mutant SIRT1 (Figure 4A). The wild-type and mutant SIRT1 was used in dual-luciferase assay, which indicated that with miR-124 overexpression using LV-miR-124, luciferase activity associated with wild-type SIRT1 (0.4 ± 0.06) was decreased compared with cells transfected with LV-Mock (1 ± 0.09, *P*<0.001). In contrast, the luciferase activity associated with mutant SIRT1 was not affected by miR-124 overexpression (Figure 4B) (0.96 ± 0.1 compared with 1 ± 0.14, *P*=0.44). In line with this, in cultured primary cortical neurons, miR-124 overexpression and down-regulation resulted in suppression and induction of SIRT1 in mRNA (0.25 ± 0.06 and 2.84 ± 0.36 for neurons with miR-124 overexpression and down-regulation, respectively, using neurons transfected with LV-Mock as controls, *P*<0.001) (Figure 4C) and protein levels (Figure 4D). Therefore, SIRT1 is a functional target of miR-124.

### SIRT1 overexpression attenuates CUMS-induced depression-like behavior

**Figure 5:**
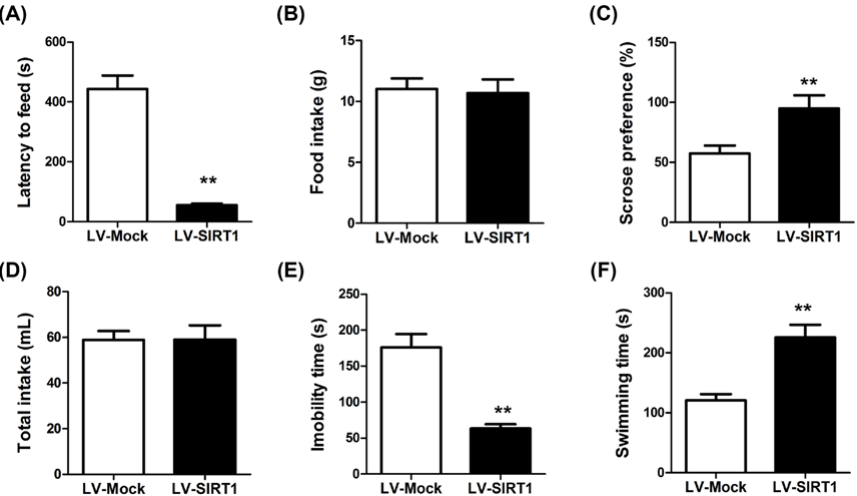
Effect of lentiviral-mediated SIRT1 overexpression on CUMS-induced depression-like behavior in the NSFT, SPT, and FST (**A**) Eating delay of the mouse as measured. (**B**) The home-cage feed consumption. (**C**) Sucrose preference and (**D**) the total fluid intake were calculated as mentioned above, (**E**) immobility and (**F**) swimming in FST of the rats. *n*=8, **P<0.01 compared with LV-Mock group.

Next, the effects of SIRT1 overexpression on CUMS-induced depression-like behavior were evaluated. After treatment with lentiviruses for SIRT1 overexpression, decrease in latency to feed (56.0 ± 4.99 s of LV-SIRT1 group compared with 443.87 ± 44.48 s of LV-Mock group, *P*<0.001, *n*=8) (Figure 5A), increase in sucrose preference (95.06 ± 10.93 of LV-SIRT1 group compared with 57.48 ± 6.52 of LV-Mock group, *P*<0.001, *n*=8) (Figure 5C), decrease in immobility time (63.41 ± 5.93 of LV-SIRT1 group compared with 176.16 ± 18.45 of LV-Mock group, *P*<0.001, *n*=8) (Figure 5E) and increase in swimming time (225.55 ± 21.11 s of LV-SIRT1 group compared with 120.53 ± 10.66 s of LV-Mock group, *P*<0.001, *n*=8) (Figure 5F). Meanwhile, no changes in food intake (Figure 5B) and total intake (Figure 5D) were observed after SIRT1 overexpression. These data underscored that high levels of SIRT1 could relieve depression.

## Discussion

With increasing understanding of the underlying molecular mechanisms that contribute to depression, there has been a continuous search for new therapeutics to suppress this psychiatric disorder. In the present study, we characterized miR-124 suppression as a therapeutic strategy for depression. The dysregulation of miR-124 in patients with depression has been previously shown [21]. However, previous studies evaluated miR-124 expression in blood serum [22] or peripheral blood mononuclear cells [21]. We for the first time reported the upregulation of miR-124 in the PFC in a mouse model of depression.

Our findings suggested that miR-124 suppression effectively reduced depression-like behaviors. Consistent with our findings, Abe-Higuchi et al. proposed that in hippocampal neurons, miR-124 overexpression confers changes in behavior phenotype to chronic ultra-mild stress, while suppression of this miRNA leads to greater behavioral predisposition to a milder stress prototype [13]. Bahi et al. reported that miR-124 overexpression intensifies depression-like behaviors, whereas miR-124 inhibition reduces depression-like behaviors in rodents induced by stress [23]. Indeed, enormous evidences supported the role of miR-124 as an important regulator of neuron development [24–26]. Our approach for administering LV-si-miR-124, i.e., through bilateral injection into PFC, presents a localized and accurate gene delivery approach, which is highly clinically translatable. Lentivirus-mediated miRNA expression is also a relatively mature technique to induce target gene expression [27]. However, further studies are needed to demonstrate whether miR-124 suppression exerts adverse side effects, given the important regulatory role of miR-124 in neuron development.

We attributed the anti-depressive behavior of miR-124 suppression to the regulation of SIRT1. The important role of SIRT1 gene in depression has been demonstrated, which leads to the hypothesis that clinical treatment of depression can be carried out by using the therapeutic drugs that directly target SIRT1 [28]. We also demonstrated that ectopic expression of SIRT1 mediated by lentiviruses was efficient in attenuating CUMS induced depression-like behaviors in mice (Figure 5). Accumulating evidences indicated that the expression of SIRT1 is mediated by miRNAs [19,20]. For example, Choi and Kemper previously showed that SIRT1 3′-UTR serves as a binding site for miR-34a [29]. In human CD4^+^ T cells, *in vitro* overexpression of miR-124a results in the blockage of SIRT1 expression [30]. Here in the present study, we demonstrated the link between miR-124 and SIRT1 in a mouse model. Since a number of other miRNAs, such miR-9, miR-146, miR-143, miR-132, miR-34a, miR-22, and miR-217, have also been shown to target SIRT1 in other diseases, it is worth investigating whether these miRNAs could serve as therapeutic targets in depression [19,31–34]. In addition, a correlation analysis between miR-124 and SIRT1 levels would be beneficial to demonstrate the link between the two genes, which was not achieved in our study due to limited number of subjects. Further studies that demonstrate the correlation between miR-124 and SIRT1 in a larger sample pool is warranted.

In conclusion, the results of the present study suggest that elevated level of miR-124 expression in PFC is associated with depression-like behaviors in a mouse model. miR-124 suppression was able to exert ameliorating effects against depression. SIRT1 is the target of miR-124. Our findings provided a potential tool for clinical management of depression using a gene therapy approach.

## Supporting information

**Supplementary Table S1:** Statistical analysis for Fig.1

**Supplementary Table S2:** Statistical analysis for Fig.2

**Supplementary Table S3:** Statistical analysis for Fig.3

**Supplementary Table S4:** Statistical analysis for Fig.4

**Supplementary Table S5:** Statistical analysis for Fig.5

## Abbreviations

CREB1: cyclic AMP responsive element binding protein 1
CUMS: chronic unpredictable mild stress
LV: lentiviral vector
PFC: prefrontal cortex
RT-PCR: real-time PCR
3′ -UTR: 3′ untranslated regions
