# Supplementary material for: MiR-124 suppression in the prefrontal cortex reduces depression-like behavior in mice
Source: Biosci Rep. 2019 Sep 13;39(9):BSR20190186. doi: 10.1042/BSR20190186 (PMC6744582; doi:10.1042/BSR20190186)
Supplement: Supplementary file 1 [file bsr20190186_Supp1.pdf]

Table 1. Statistical analysis for Fig.1

Factor	Group	mean±SD	df	F	P value	t
miR-124	0	1.00±0.15	5	55.88	<0.001	-
	1	1.10±0.16				0.6657
	2	1.16±0.23				1.027
	3	2.15±0.23***				7.786
	4	2.59±0.32***				10.73
	5	2.68±0.38***				11.32
SIRT1	0	1.00±0.15	5	38.16	<0.001	-
	1	0.90±0.11				1.525
	2	0.95±0.18				0.6887
	3	0.54±0.08***				6.739
	4	0.40±0.06***				8.929
	5	0.34±0.06***				9.716

Bonferroni posttests were used when F values were significant, t test get from the group compared with 0 group

Table 2. Statistical analysis for Fig.2

751±71.79 s vs. 439±32.02 s

factor	group	mean±SD	df	F	p	T(Compare with LV-Mock)
latency to food(s)	LV-Mock	439±32.02				-
	LV-miR-124	751±71.79	2	293.600	<0.001	11.81
	LV-si-miR-124	65±6.28				12.43
food intake(g)	LV-Mock	9±1.2				-
	LV-miR-124	11±1.16	2	2.413	0.123	2.179
	LV-si-miR-124	10±0.99				1.328
Sucrose preference	LV-Mock	53±5				-
	LV-miR-124	22±3.35	2	169.000	<0.001	8.15
	LV-si-miR-124	92±9.52				10.2
total intake(mL)	LV-Mock	64±5.76				-
	LV-miR-124	61±4.19	2	0.606	0.558	1.099
	LV-si-miR-124	63±7.08				0.5014
immobility time(s)	LV-Mock	170±16.61				-
	LV-miR-124	242±25.04	2	186.900	<0.001	7.281
	LV-si-miR-124	47±7.26				11.87
Swimming time(s)	LV-Mock	120±11.65				-
	LV-miR-124	65±6.28	2	131.500	<0.001	4.616
	LV-si-miR-124	247±31.89				11.16

Bonferroni posttests were used when F values were significant, t test get from the group compared with LV-Mock group

Table 3. Statistical analysis for Fig.3

factor	group	mean±SD	df	F	p	t(Compare with LV-Mock)
miR-124	LV-Mock	1±0.15	2	187.80	<0.001	-
	LV-miR-124	3.4±0.47				14.56
	LV-si-miR-124	0.36±0.07				3.795
sirt1	LV-Mock	1±0.16	2	246.10	<0.001	-
	LV-miR-124	0.3±0.05				5.838
	LV-si-miR-124	2.7±0.31				15.62
BDNF	LV-Mock	1±0.11	2	163.30	<0.001	-
	LV-miR-124	0.4±0.06				5.542
	LV-si-miR-124	2.3±0.3				12.12
CREB1	LV-Mock	1±0.14	2	126	<0.001	-
	LV-miR-124	0.5±0.07				5.701
	LV-si-miR-124	1.9±0.21				9.981
pCREB1	LV-Mock	1±0.07	2	64.52	<0.001	-
	LV-miR-124	2.00				4.886
	LV-si-miR-124	64.52				6.439

Table 4. Statistical analysis for Fig.4

factor		group	mean±SD	df	F	p	t(Compare with LV-Mock)
Luciferase activity	SIRT1 3'UTR-wt	LV-Mock	1±0.09	2	171.40	<0.001	
		LV-miR-124	0.4±0.06				
	SIRT1 3'UTR-mut	LV-Mock	1±0.14	2	0.44	0.52	
		LV-miR-124	0.96±0.1				
SIRT1 Mrna expression		LV-Mock	1±0.11	2	217.00	<0.001	-
		LV-miR-124	0.25±0.06				5.849
		LV-si-miR-124	2.84±0.36				14.39

Table 5. Statistical analysis for Fig.5

		N	mean	sd	df	F	p
latency to food(s)	LV-Mock	6	443.87	44.48	1	451	<0.001
	LV-SIRT1	6	56.00	4.99			
food intake(g)	LV-Mock	6	11.03	.85	1		1
	LV-SIRT1	6	10.68	1.13			
Sucrose preference	LV-Mock	6	57.48	6.52	1	52	<0.001
	LV-SIRT1	6	95.06	10.93			
total intake(mL)	LV-Mock	6	58.86	3.94	1		1
	LV-SIRT1	6	59.01	6.20			
immobility time(s)	LV-Mock	6	176.16	18.45	1	203	<0.001
	LV-SIRT1	6	63.41	5.93			
Swimming time(s)	LV-Mock	6	120.53	10.66	1	118	<0.001
	LV-SIRT1	6	225.55	21.11			
